# Investigation of multidrug-resistant fatal colisepticaemia in weanling pigs

**DOI:** 10.4102/ojvr.v82i1.986

**Published:** 2015-11-27

**Authors:** Folorunso O. Fasina, Dauda G. Bwala, Evelyn Madoroba

**Affiliations:** 1 Department of Production Animal Studies, University of Pretoria, South Africa; 2 Bacteriology Section, Agricultural Research Council–Onderstepoort Veterinary Institute, South Africa

## Abstract

*Escherichia coli* is usually a benign commensal of the gut microflora. However, when *E. coli* acquires virulence genes it can multiply rapidly and cause disease through colonisation of the intestinal mucosa. *Escherichia coli* can become a significant pathogen in young pigs. We report an investigation of fatal colisepticaemia in weanling pigs from emerging farms where piglets and weaners were diarrhoeic and the mortality rate ranged between 15% and 70% in each litter. Faecal and tissue samples were processed for histopathology, bacteriology and molecular biology (multiplex and monoplex polymerase chain reaction) and we recovered enteroaggregative multidrug-resistant *E. coli* producing *EAST*-*1* enterotoxin. An association between poor housing conditions and the observed cases was established and future management programmes were recommended to reduce the impact of such pathogens. Enteroaggregative *E. coli* is becoming a major problem in the pig industry. It therefore becomes necessary to establish the full impact of *E. coli* on the South African pig industry and to determine the geographic extent of the problem.

## Communication

Rotavirus, *Escherichia coli, Clostridium perfringens, Isospora suis*, transmissible gastro-enteritis virus and *Enterococcus durans* are amongst the most common diarrhoea-causing pathogens in piglets and are sometimes associated with neonatal and weanling deaths (Johnson *et al*. 1992; Martins *et al*. 2000; Vu-Khac, Holoda & Pilipcinec 2004). *Escherichia coli* is generally a benign commensal of the gut microflora. However, when the bacterium acquires virulence genes it can multiply rapidly and colonise the intestinal mucosa by using surface proteins (fimbriae). The subsequent production of heat-stable or heat-labile toxins causes disease (Parma *et al*. 2000). Diarrhoea caused by *E. coli* affects all categories of young pigs (piglets, weaners and growers) to different degrees (Henton & Engelbrecht 1997; Nagy & Fekete 1999; Vu-Khac *et al*. 2006).

Recent reports have indicated that prevalence and isolation of antimicrobial-resistant *E. coli* are on the increase (Enne *et al*. 2008; Luppi *et al*. 2015; Toledo *et al*. 2012). Post-weaning diarrhoea (PWD) is a recurrent problem in weaned pigs (3–4 weeks of age) and previous studies have associated this condition with the F4 (mainly K88ac) and F18 fimbriae virulence factors (Nagy & Fekete 1999; Vu-Khac *et al*. 2006). In South Africa specifically, Henton and Engelbrecht (1997) have serotyped 674 isolates from pigs and found that types O149, O141, O9, O20, O8 and a few others are prevalent in South Africa (arranged here in descending order of prevalence). F4 was associated with 46.9% of the isolates. Similar reports have been obtained from other smallholder farms and emerging pig farms despite slight variations in the pattern of presentation.

In December 2013, carcasses of two five-week-old Large White–Landrace cross weanlings were presented to the Pathology Laboratory of the Faculty of Veterinary Science, University of Pretoria (accession number S04444-13). Earlier, a report based on routine visits of the Porcine Herd Health team to the farm of origin (a start-up farm with 60 sow units) indicated that certain design and management errors had predisposed piglets in the farrowing unit to wet floors, cold draughts and hypothermia and the associated consequences. Suggestions for correction were offered. By the time the carcasses were presented for post-mortem examination, 14 sows had produced an average of 12 piglets per litter, of which 11 per litter were weaned at 4 weeks (weaning weight ≈ 6.7 kg; *n* = 151). PWD and mortality started 3–7 days post weaning and continued for the next 2 weeks, ultimately becoming self-limiting. Approximately 80% of the piglets experienced PWD to some extent. The mortality rate varied from 15% to 70% in affected litters.

Necropsy revealed severe generalised congestion, severe segmental catarrhal enteritis, moderate nephrosis and mild acute hepatosis. Generalised lymphadenomegaly due to cortical hyperplasia and diffuse moderate interstitial pneumonia with atelectasis were also observed.

**FIGURE 1:**
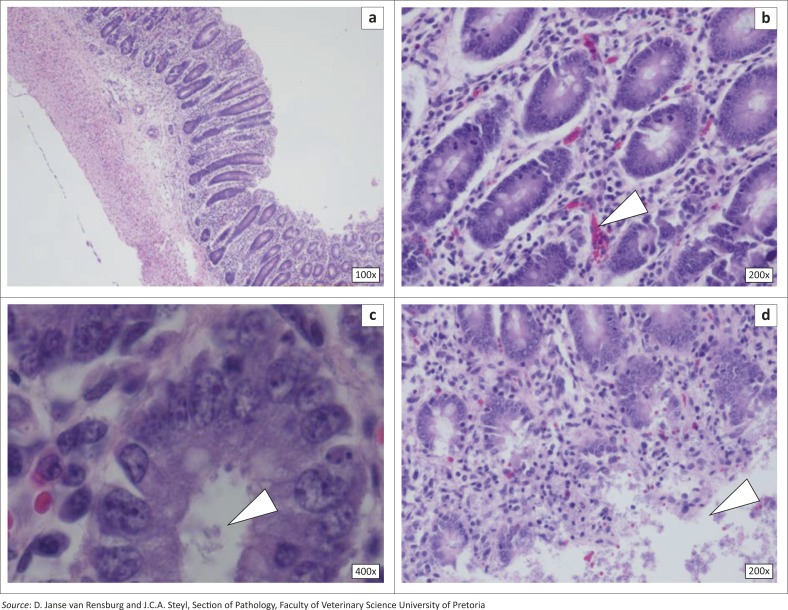

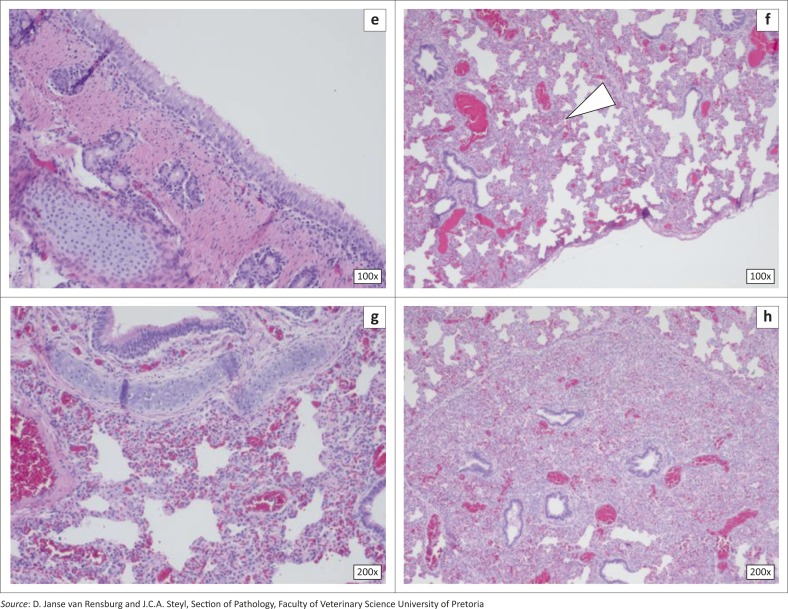
Histopathology of the intestinal and respiratory system tissue from the studied animals, stained with haematoxylin–eosin. (a) Overview of a transverse section of intestine; (b) Intestinal lumen, with normal lymphocytes and plasma cells in the lamina, mild congestion of enterocytes and lymphoid cells (arrow) and normal mucosal cells; (c) Lumen filled with bacteria and debris (arrow), normal enterocytes and goblet cells; (d) Section of the intestine, showing normal enterocytes, congestion, mild haemorrhage and autolytic cells (arrow); (e) Transverse section of the pig trachea showing the respiratory epithelium, blood vessels and hyaline cartilage; (f) Interstitial alveolar wall thickening (arrow); (g) Interstitial pneumonia and congestion; (h) Consolidated lung tissue with numerous bronchioles and congestion.

Multifocal villus crypts filled with a monopopulation of small bacterial rods histologically typical of *E. coli* were observed in tissue samples collected for histopathology (Figure 1a–c). The rod-like organisms adhered to enterocyte brush borders and were scattered within the lamina propria, extending to the lamina muscularis of the small intestine. The intestinal mucosa was not well differentiated and goblet cells were sparse, with a high mitotic rate (Figure 1d), indicative of regeneration and repair.

Multifocal atelectasis was observed in the lungs, interspersed by areas of marked interstitial pneumonia. Large numbers of mononuclear cells, mainly macrophages and lymphocytes, had infiltrated and distended the alveolar walls (Figure 1e–h). Multifocal areas of protein-rich alveolar oedema were observed. The lymph nodes showed numerous lymphoblasts within follicles, with prominent medullary congestion.

Based on the presumptive aetiological diagnosis, diarrhoeic faeces were collected from sick piglets during a follow-up visit to the farm. Culture on blood agar revealed both rough and smooth non-haemolytic *E. coli* organisms (pathology laboratory accession number B04025-14). During an *in-vitro* antimicrobial sensitivity evaluation, these organisms were found to be resistant to ampicillin, kanamycin, trimethoprim–sulphadimidine, oxytetracycline and tylosin, but susceptible to cetriofur, enrofloxacin and florfenicol. Subsequent faecal samples collected between January 2014 and June 2015 from this farm and others with matching production statuses revealed similar patterns of clinical signs, pathology and bacterial cultures. However, variable patterns of antibiotic resistance were observed, based primarily on the predominant antibiotics used on the farms.

All isolates were subcultured on blood agar and molecular characterisation was conducted. For this purpose, we conducted an investigation into the virulence factors associated with PWD using standardised protocols for polymerase chain reaction (PCR) and previously determined primers (Table 1).

**TABLE 1:** Primer sequences of *Escherichia coli* virulence factors tested in the study.

Virulence factor	Nucleotide sequence (5′-3′)	Size (bp)	Target gene	Reference
Sta-F	GGG TTG GCA ATT TTT ATT TCT GTA	183	*estI*	Cai *et al.* 2003; Ngeleka *et al*. 2003
Sta-R	ATT ACA ACA AAG TTC ACA AGC AGT A	-	-	-
STb-F	ATG TAA ATA CCT ACA ACG GGT GAT	360	*estII*	-
STb-R	TAT TTG GGC GCC AAA GCA TGC TCC	-	-	-
LT-F	TAG AGA CCG GTATTA CAG AAATCT GA	282	*elt*	Cai *et al.* 2003; Ngeleka *et al*. 2003
LT-R	TCA TCC CGA ATT CTG TTA TAT ATG TC	-	-	-
EAST-1-F	TCG GAT GCC ATC AAC ACA GT	125	*astA*	Cheng *et al.* 2006
EAST-1-R	GTC GCG AGT GAC GGC TTT GTA G	-	-	-
Stx1-F	ATT CGC TGA ATG TCATTC GCT	664	*stxI*	Cai *et al.* 2003
Stx1-R	ACG CTT CCC AGA ATT GCA TTA	-	-	-
Stx2-F	GAA TGA AGA AGA TGT TTA TAG CGG	281	*stxII*	Cai *et al.* 2003
Stx2-R	GGT TAT GCC TCA GTC ATT ATT AA	-	-	-
Stx2e-F	GAA TGA AGA AGA TGT TTA TAG CGG	454	*stx2e*	Cai *et al.* 2003
Stx2e-R	TTT TAT GGA ACG TAG GTA TTA CC	-	-	-
AIDA-1-F	ACA GTA TCA TAT GGA GCC A	585	*aidA*	Cheng *et al.* 2006
AIDA-1-R	TGT GCG CCA GAA CTA TTA	-	-	-
EAE-F	CAT TAT GGA ACG GCA GAG GT	790	*eae*	Cheng *et al.* 2006
EAE-R	ATC TTC TGC GTA CTG CGT TCA	-	-	-
PAA-F	ATG AGG AAC ATA ATG GCA GG	360	*paa*	Cheng *et al.* 2006
PAA-R	TCT GGT CAG GTC GTC AAT AC	-	-	-
F4 (K88)-F	GAT GAA AAA GAC TCT GAT TGC A	841	*faeG*	Cai *et al.* 2003; Ngeleka *et al*. 2003
F4 (K88)-R	GAT TGC TAC GTT CAG CGG AGC G	-	-	-
F5 (K99)-F	CTG AAA AAA ACA CTG CTA GCT ATT	543	*fanA*	Cai *et al.* 2003; Ngeleka *et al*. 2003
F5 (K99)-R	CAT ATA AGT GAC TAA GAA GGA TGC	-	-	-
F41-F	GAT GAA AAA GAC TCT GAT TGC A	682	*fim41a*	Cai *et al.* 2003; Ngeleka *et al*. 2003
F41-R	TCT GAG GTC ATC CCA ATT GTG G	-	-	-
F6 (987P)-F	GTT ACT GCC AGT CTA TGC CAA GTG	463	*fasA*	Cai *et al.* 2003; Ngeleka *et al*. 2003
F6 (987P)-R	TCG GTG TAC CTG CTG AAC GAA TAG	-	-	-
F18-F	ATG AAA AGA CTA GTG TTT ATT TCT T	513	*fedA*	Ngeleka *et al.* 2003
F18-R	TTA CTT GTA AGT ACC GCG TAA GCC	-	-	-

Note: Please see the full reference list of the article, Fasina, F.O., Bwala, D.G. & Madoroba, E., 2015, ‘Investigation of multidrug-resistant fatal colisepticaemia in weanling pigs’, *Onderstepoort Journal of Veterinary Research* 82(1), Art. #986, 6 pages. http://dx.doi.org/10.4102/ojvr.v82i1.986, for more information.

bp, base pair.

*Escherichia coli* DNA was extracted using cell lysis. Bacterial cells were boiled at 99 °C for 15 min, followed by centrifugation. The supernatant containing crude DNA extracts was used in multiplex and monoplex PCR reactions targeting the following virulence factors: heat-labile toxin (*LT*), heat-stable toxin A (*STa*), heat-stable toxin B (*STb*), shiga toxins *Stx1, Stx2* and *Stx2e*, enteroaggregative heat-stable enterotoxin (*EAST*-1), adhesin involved in diffuse adherence 1 (*AIDA-1*), porcine attaching- and effacing-associated factor (*PAA*) and fimbriae F4, F5, F6, F41 and F18. Primers used in the multiplex and monoplex PCR reactions were combined as described by Mohlatlole *et al*. (2013). The primers that were used for DNA amplification in the PCR reactions are listed in Table 1. The composition of the 25-µL PCR reactions was as follows: 2.5 µM of each primers, 5 µL crude DNA extract, 12.5 µL PCR Master Mix (Fermentas) and sterile DNAse-free water (Fermentas). The conditions for amplification were as follows: 10 min initial denaturation at 94 °C, followed by 30 cycles of denaturation at 94 °C for 30 s, annealing at 56 °C for 30 s and extension at 72 °C for 1 min. To ensure complete amplification of the PCR products, the thermocycling conditions included 7 min of extension at 72 °C.

Monoplex PCR reactions were set up to confirm EAST toxins in the samples. Similar PCR amplification conditions were used, except for the volume of water being adjusted to a final volume of 25 µL using sterile DNAse-free water.

Reference and control samples were included for all PCR reactions. For this purpose, reference samples that are known to be positive for enterotoxins and fimbriae were obtained from the Culture Collection of the Bacteriology Section of the Agricultural Research Council – Onderstepoort Veterinary Institute (ARC–OVI). The strains included B41 (F5:F41:STa), 1883–1 (F18:STa), 1883–2 (F41:F5:STa), 1883–3 (F41:F5:F6:STa), 1883–4 (F4:F5:LT:STb), 1474 K12-K99 Pienk (F5:STa), 1474 K12-K99 Geel (F5:STa) and K99 (F5:STa). In addition, isolates that were known to be positive for *EAST*-1, PAA, AIDA-1 and *E. coli* attaching and effacing (EAE) factor reference samples were included. *E. coli* ATCC 25922 was used as the negative control for virulence factors.

**FIGURE 2:**
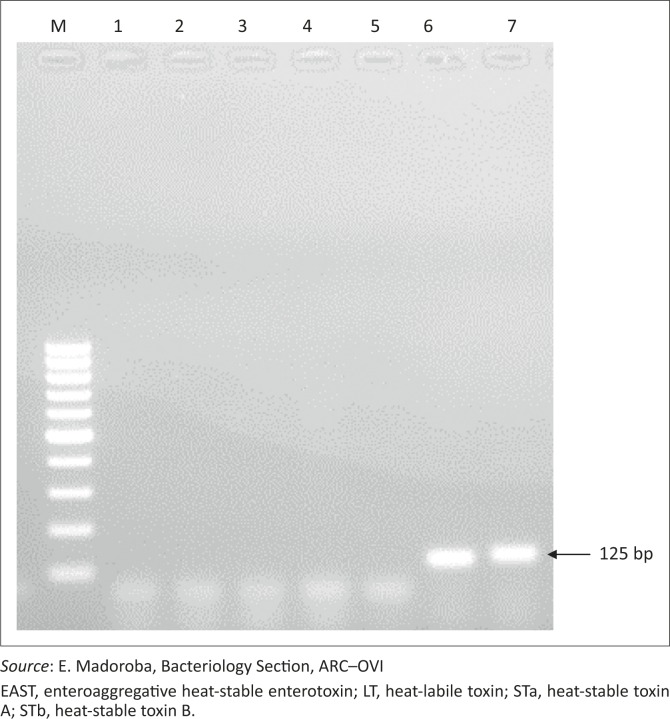
Gel electrophoresis showing *EAST*-1 positive amplicons. Lanes: 1, 100-bp plus DNA ladder; 2, STb negative; 3, LT negative; 4, *E. coli* ATCC 25922 negative control strain; 5, STa negative; 6 and 7, EAST positive.

The PCR results indicated that of the 15 virulence factors tested, only *EAST*-1 yielded a positive band. Although most outbreaks of *E. coli*-derived diarrhoea in animals have been associated with strains with more than one virulence factor, in this case only *EAST*-1 toxin was observed (both *E. coli* isolates were positive for the 125-base-pair toxin; see Figure 2). EAST toxins, produced by enteroaggregative *E. coli*, are antigenically related to the heat-stable toxins produced by enterotoxigenic *E. coli* and are known to induce fluid secretion (Veilleux & Dubreuil 2006). Nevertheless, the products encoded by *EAST* have also been found to be both diarrhoeagenic and non-pathogenic (Ruan *et al*. 2012). Although our results confirmed that *E. coli* was involved in causing disease and the associated mortalities in this case, involvement of other organisms was not explored. The *EAST*-1 toxins have been found in South African pigs in a previous study (Mohlatlole *et al*. 2013), which reported that 22.5% of the *E. coli* isolates carried the *EAST*-1 gene. Similar results have been obtained in studies from Korea and the Czech Republic, although higher prevalences were obtained in sucklers compared with weaner pigs (Lee *et al*. 2008; Zajacova, Konstantinova & Alexa 2012). As the *EAST*-1 toxin could exacerbate the pathogenesis of *E. coli* diarrhoea in weaned pigs (Choi *et al*. 2001), it will be necessary to determine the spatio-temporal extent of these organisms in the South African pig population to understand the epidemiology of the infection in the country. Furthermore, no *E. coli* vaccine available in South Africa confers protection against the *EAST*-1 toxin, which may partially explain the high mortality observed in some of the cases.

Based on the outcome of the pathology and PCR results, a conclusive diagnosis of colisepticaemia was made. At weaning, piglets are subjected to a variety of stress factors, including the withdrawal of the dam and sow's milk, a dip in the concentration of CD4^+^ and CD8^+^ cells, neutrophils and lymphocytes, and a post-weaning syndrome that consists of PWD, oedema disease and endotoxin shock (Almond & Kirk 2010; Van Beers-Schreurs *et al*. 1992). The change in gut microflora associated with the changing diet around the time of weaning may lead to *E. coli* overgrowth and subsequent enteritis and endotoxaemia. On this farm, several cases of diarrhoea were observed in the farrowing and weaner pens and respiratory distress was observed in young weaners. Although the *E. coli*-associated enteritis could have compromised the integrity of the intestinal villi and reduced the efficiency of feed utilisation, the cause of death in these weaner pigs was *E. coli*-associated endotoxaemia. No cases of piglet anaemia were observed in the herd and the oxygen-carrying capacity of haemoglobin did not appear compromised. The consolidation and interstitial alveolar wall thickening observed in the lungs notably reduced the capacity of the lungs in complying with their primary role of oxygen–carbon dioxide exchange at the alveolar level. Fast-growing weaner pigs need an increasing level of such gaseous exchange to meet their physiologic needs at this stage. It becomes vitally important to reduce other associated complications (e.g. digestive or respiratory) arising from *E. coli* infection at the time of weaning.

We recommended changes in management protocol at this farm to ensure dry floors and hygiene in the farrowing and weaner units. Establishment of a vaccination protocol that includes routine *E. coli* vaccination of sows 2 weeks before parturition is also recommended to boost colostral immunity and decrease the magnitude of antibiotic treatment in subsequent outbreaks.
